# Interactions between Host PPARs and Gut Microbiota in Health and Disease

**DOI:** 10.3390/ijms20020387

**Published:** 2019-01-17

**Authors:** Arif Ul Hasan, Asadur Rahman, Hiroyuki Kobori

**Affiliations:** Department of Pharmacology, School of Medicine, International University of Health and Welfare, 4-2 Kozunomori, Narita, Chiba 286-8686, Japan; hasan@iuhw.ac.jp (A.U.H.); rahmanma@iuhw.ac.jp (A.R.)

**Keywords:** atherosclerosis, inflammatory bowel disease, irritable bowel syndrome, metabolic syndrome, non-alcoholic liver diseases, obesity, type 2 diabetes mellitus

## Abstract

The human gastrointestinal tract is inhabited by many types of microbiota, including bacteria, viruses, and fungi. Dysregulations of their microenvironment are associated with various health problems, not only limited to gastrointestinal disorders, such as inflammatory bowel disease, but to impacts beyond the intestine. For example, intestinal microbiota can affect the liver in non-alcoholic fatty liver disease, visceral adipose tissue during adipogenesis, and the heart in atherosclerosis. The factors contributing to these pathogeneses involve the gut microbiota and the effector organs of the host, and everything in between. The nuclear receptor peroxisome proliferator-activated receptors (PPARs) are pivotal for the modulation of many of the pathogeneses mentioned above. It is, therefore, conceivable that, in the process of host-microbiota interactions, PPARs play important roles. In this review, we focus on the interactions between host PPARs in different organs and gut microbiota and their impacts on maintaining health and various diseases.

## 1. Introduction

Each of us hosts trillions of lives along with us, remaining unnoticed most of the time. These lives are the bacteria, archaea, and viruses residing on the surface of our body and inside the gut [1]. The gut-dwelling microbes are influenced by the part of the globe in which we live, the type of foods we eat, our age, how we were born, and many more factors [1,2,3]. The skin microbial community is influenced by external factors such as climate and hygiene, as well as internal factors, such as physiology and disease state [1]. As a result, the microbial community we host, both inside and out, is exclusive to each individual [3].

Are these bacteria our friends or foes? Do these host-specific diversities in the bacterial population have any impact on our health? Answers to these questions reside inside us. Any of the gut- or skin-dwelling microbial populations have potential impacts on our health. However, for the purposes of this review, we confine our discussion to the bacterial communities in the human intestine. The intestinal microbes reside in our hostile but nutrition-rich gastrointestinal tract at the cost of helping us digest complex forms of foods. All these microbes inhabiting within us together encode over three million genes, which is 150 times of the number of human genes [4]. Utilizing these genes, the microbes produce short-chain fatty acids (i.e., butyrate, propionate, etc.) [5], ligands for G-protein-coupled receptors (i.e., *N*-acyl amide) [6], neurotransmitters (i.e., serotonin, dopamine, etc.) [7], and other metabolites. These metabolites genetically and epigenetically influence host responses [2].

Despite these beneficial effects, we are always at risk of infection [8]. Therefore, our gut epithelium tries to protect us by not allowing these microbes and their harmful products to enter our system. Scientists provided compelling evidence of the relationship between some diseases and disequilibrium in the microbiota. These diseases include type 2 diabetes mellitus, non-alcoholic fatty liver diseases, irritable bowel syndrome, and inflammatory bowel diseases [9]. Most of these incurable diseases are a socio-economic burden for many countries.

From the historical perspective of gut-centered disease, possibly pioneered by the view of the “father of modern medicine”, Hippocrates, “all disease begins in the gut”. Indeed, fecal microbiota transplantation was successfully used to treat inflammatory bowel diseases and irritable bowel syndrome, and is thought to have roles in type 2 diabetes and metabolic diseases [10,11]. Details of the beneficial or adverse effects of these host–microbiota interactions were reviewed elsewhere. However, in this vast field, some interesting interactions occur between a group of transcriptional factors, called peroxisome proliferator-activated receptors (PPARs), with gut microbiota, which we review here.

In humans, the nuclear receptor superfamily includes 48 transcriptional factors. They are activated by their specific ligands, and regulate diverse developmental, inflammatory, and metabolic processes [12,13]. PPARs are members of this nuclear receptor superfamily. PPARs are composed of three members: PPARα, PPARβ/δ, and PPARγ, also known as nuclear receptor subfamily 1, group C, members (NR1C)-1, -2, and -3, respectively. PPARα was the first member identified in the early 1990s, as a target of hypolipidemic fibrate [14]. Based on their sequence homology, PPARβ/δ and PPARγ were cloned [15,16]. Due to their close association with obesity and additional cardiovascular complications, these receptors were extensively studied since their identification [17,18].

PPARα is predominantly expressed in the liver, as well as in the heart, muscle tissue, kidney, and brown adipose tissue [17,19]. PPARα is mostly involved in β-oxidation and fatty-acid transportation and, thus, controls lipid homeostasis [20]. PPARβ/δ is expressed in skeletal muscle, the gastrointestinal tract, heart, brown adipose tissue, and white adipose tissue. The high activity of PPARβ/δ in skeletal muscle regulates fatty-acid catabolism. In adipose tissue, it improves lipid metabolism. PPARγ is expressed in brown adipose tissue, white adipose tissue, the colon, and immune cells. In brown adipose tissue, it causes browning of adipocytes and improves thermogenesis. Several major functions of PPARγ include uptake and safe deposition of lipids in adipose tissue, liver, and muscle; regulation of adipocytokine secretion; and improving insulin resistance [17,20].

Given their widespread distribution in various organs, the effects of PPARs are not limited to the abovementioned conditions. Despite the spatial barrier between the PPARs of some organs, such as the liver, adipose tissue, and the intestinal microbiota, they interact with each other. Briefly, the metabolites produced by the microbiota are absorbed by the intestinal epithelial and resident inflammatory cells. The metabolites are also transported to liver, adipose tissue, heart, blood vessels, and other organs through systemic circulation. In these organs, the metabolites act as ligands of PPARs. Activation of PPARs modulate (1) intestinal, as well as the whole-body immune response, and (2) carbohydrate and fat metabolism. From these perspectives, we elaborate upon the molecular mechanisms related to PPARs and gut microbiota in health and some diseases.

## 2. Gut Microbiota Composition

In healthy humans, the number of bacteria in the intestine was previously considered to be around 1 × 10^14^ [21]. According to a recent estimation, the number is thought to be around 3.8 × 10^13^ [22]. As the amount of bacteria is more than 1000-fold lower in the small intestine, the counts of intestinal bacteria mentioned above mostly represent bacteria in the large intestine [22]. The sequencing studies targeting 16s rRNA (small subunit ribosomal RNA) showed that most of these intestinal bacteria fall under four phyla: Firmicutes, Bacteroidetes, Actinobacteria, and Proteobacteria [23]. Each phylum contains many different species [3], resulting in between 1000 and 1150 prevalent bacterial species [4]. However, only about 160 of these species are shared among human subjects [4].

The two predominant phyla are Firmicutes, which includes *Lactobacillus*, *Clostridium*, *Enterococcus*, and *Ruminococcus*, and Bacteroidetes, which includes *Prevotella* and *Bacteroides* genera. In children of Burkina Faso, a West African country, the proportions of Firmicutes and Bacteroidetes were 12% and 73%, respectively, whereas these proportions in children from Florence, Italy were 51% and 27%, respectively [24]. Differences in dietary habit are considered to shape the bacterial populations in the children from these two geographical areas. Similarly, exclusively formula-fed infants are more colonized with *Escherichia coli*, *Clostridium difficile*, *Bacteroides fragilis*, and *Lactobacilli* than exclusively breastfed infants [25]. In vaginally delivered infants, *Clostridium* (*C. difficile*) and *Lactobacillus* genera are more common compared with infants delivered via caesarean section [25]. The abundance and diversity of the phyla Actinobacteria and Bacteroidetes are lower in caesarean-section-delivered infants. However, the diversities at 6 to 12 months are similar irrespective of delivery mode [26]. The diversity again appears in patients with various diseases such as type 2 diabetes mellitus, metabolic syndrome, etc. [9]. These observations suggest the existence of host-specific diversities among the bacteria, those that can affect or be affected by the host.

## 3. PPARs on Microbial Inhabitation and Adaptation in the Gut

For colonization and survival in a specific niche, microbiota modulate the expression of PPARs in intestinal epithelial and immune modulatory cells and alter the host inflammatory responses. Therefore, cross-talk between the commensal microbiota and the host cell signaling molecules must start immediately after birth. *Enterococcus faecalis* is an early colonizer, transferred from mother to child. *E. faecalis*, when isolated from newborn babies co-cultured with human colorectal adenocarcinoma cell line (HT-29) or murine epithelial cells, showed enhanced PPARγ1 phosphorylation. This phosphorylation also elevated DNA binding of PPARγ1 and its transcriptional activation of an innate immune system modulator, interleukin (IL)-10 [27]. IL-10 binds with the IL-10 receptor expressed in the macrophages, and polarizes the macrophages to an anti-inflammatory phenotype, namely C–X–3–C motif chemokine receptor (CX3CR^hi^) [28]. These macrophages harness intestinal immune responses to a level suitable for maintaining gut defense without interfering with gut microbial homeostasis. From the opposite perspective, the pathogenic *Salmonella typhimurium* downregulates PPARγ expression and initiates a local inflammatory response in the intestine. This inflammatory response is hostile for the commensals; thus, this pathogen enables its own colonization [29]. Thus, PPARγ-mediated regulation of inflammatory cytokines allows commensal or pathogenic bacteria to colonize the human gut.

However, a contrasting pattern of PPARγ expression was demonstrated for *Streptococcus salivarius.* Among the commensal intestinal microbiota, *S. salivarius* is an early colonizer ubiquitously preset in the small and large intestine, with an ileac predominance [30]. In some human epithelial cell lines (HT-29, Caco-2, and SW-116), supernatants collected from *S. salivarius* reduce an inflammatory mediator: nuclear transcription factor κB (NF-κB) [31]. In this report, expression of PPARγ and two of its target genes (intestinal fatty-acid binding protein and angiopoietin-like protein 4) was found to be reduced [31]. Although no direct effect of reduced PPARγ expression on NF-κB downregulation was demonstrated in this in vitro model, the authors assumed that PPARγ-mediated suppression of inflammatory responses facilitates *S. salivarius* colonization of the intestine. In a mouse model, short-chain fatty acids (explained below), which are ligands of PPARs [5], cause expansion and differentiation of regulatory T lymphocytes (known as T_reg_ cells). This process limits pro-inflammatory responses and sustains tolerance of commensals [32].

**Figure 1:**
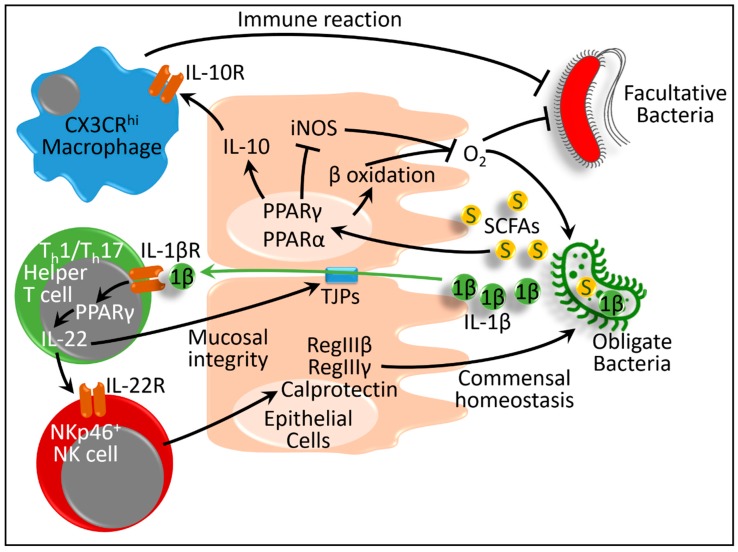
Schematic presentation of interactions between host peroxisome proliferator-activated receptors (PPARs) and gut microbiota in microbial inhabitation and adaptation. IL: interleukin; IL-1βR, IL-10R and IL-22R: receptors of interleukin -1β, -10 and -22, respectively; iNOS: inducible nitric oxide synthase; PPAR: peroxisome proliferator-activated receptors (α and γ); S and SCFAs: short-chain fatty acids; TJPs: tight junction proteins. Black lines ending in arrowheads denote activation and lines ending in bars represent inhibition. Green arrow depicts absorption of interleukin 1β.

Some of the obligate microbes have the ability to protect intestinal mucosa. Dextran sodium sulfate is a chemical compound that increases intestinal permeability and causes colitis-like effects [33]. Dextran sodium sulfate-induced colitis mice, treated with *Lactobacillus paracasei* B21060, causes upregulation of PPARγ and β-defensin. This upregulation is associated with restoration of intestinal integrity. This study suggests that the microbiota influences intestinal PPARγ in maintaining intestinal mucosal homeostasis (Figure 1) [34].

The obligate gut microbiota ferment complex foods and produce several short-chain fatty acids, namely butyrate, acetate, and propionate. Among them, butyrate is the main carbon source for the intestinal epithelial cells [35]. PPARγ responds to butyrate and drives the energy metabolism of these cells toward β-oxidation, and suppresses synthesis of inducible nitric oxide synthase (iNOS). Thereby, the oxygen bioavailability in the colon decreases. As a result of this PPARγ signaling, the anaerobic milieu in the colon is maintained, which prevents growth of facultative anaerobes [36]. However, the microbes differentially produce metabolites; thus, they differentially modulate host epithelial responses. For example, in an ex vivo model, short-chain fatty-acid-induced conditioned medium collected from *Akkermansia muciniphila* affected expression of 1005 genes in intestinal organoids, whereas *Faecalibacterium prausnitzii* affected only 503 genes. Among those, PPARγ expression was reduced by the former, whereas the latter showed no effect [37]. The authors also demonstrated that the physiological concentration of butyrate and propionate, but not acetate, modulated PPARγ and angiopoietin-like protein 4 expressions by the *A. muciniphila* [37].

In addition to PPARγ, PPARα is important for regulating commensal bacterial homeostasis. Microbiota, in particular the Clostridia-related segmented filamentous bacteria (SFB), produce IL-1β, which activates T helper 1 and 17 (T_h_1 and T_h_17) cells in the intestine [38,39]. T_h_1 and T_h_17 cells are types of cluster of differentiation 4 positive (CD4^+^) T-helper lymphocytes. They are important for protecting the intestine during enteric infection [39]. T_h_1 and T_h_17 lymphocytes express several proinflammatory cytokines such as IL-17A, IL-17F, and IL-22, which are critical for host defense and autoimmunity [40]. For example, IL-22, produced by a type of natural killer (NK) lymphocyte—NKp46^+^ innate cells—regulates intestinal immune responses [41]. This cytokine influences the expression of antimicrobial peptides (RegIIIβ, RegIIIγ, and calprotectin) via the epithelial cells to maintain the bacterial niche. RegIIIγ binds to the peptidoglycan surface of Gram-positive bacteria, such as those in the Lactobacillacae family, and confines them within the small intestine but not the colon [42]. IL-22 also maintains epithelial cell barrier integrity and helps in mucous production and epithelial cell regeneration [42,43]. Through these processes, IL-22 restores commensal homeostasis. Therefore, the absence of IL-22 increases the susceptibility to pathogenic microbiota [41,42]. In this context, in PPARα knock-out mice, the absence of PPARα produced an enhanced inflammatory response even in response to commensal bacteria. As a result, the T_h_1 and T_h_17 cells in the intestine increased. However, due to lack of PPARα overall production of IL-22, RegIIIβ and RegIIIγ decreased. As a result, dysbiosis occurred [44].

Taken together, although PPAR expression in the intestine or immune cells was not evaluated in all the abovementioned studies, it is conceivable that microbial alteration of PPAR expression, along with its target genes, facilitates intestinal homeostasis for inhabitation and adaptation of the microbes. Considering contradictory findings, the mechanisms mostly involve (1) production of inflammatory cytokines, (2) maintenance of intestinal mucosal homeostasis and integrity, and (3) modulation of immune cells (Figure 1).

## 4. Gut Microbiota and PPARs in Diseases

### 4.1. Gut Microbiota and PPAR Interaction in Gastrointestinal Diseases

Irritable bowel syndrome is characterized by abdominal discomfort or pain and alteration of bowel habits [45]. Among several proposed origins, altered bacterial flora in the pathogenesis of irritable bowel syndrome is quite compelling. Children [46] and adults [47] suffering from irritable bowel syndrome show an increased ratio of Firmicutes to Bacteroidetes. Particularly, two members of Firmicutes, *Dorea* and *Ruminococcus*, are abundantly present in these patients. *Dorea* is capable of producing formic acid [48], and *Ruminococcus* (*Ruminococcus torques*) is associated with producing greater pain severity [49]. In irritable bowel syndrome patients who experience alternating constipation and diarrhea (i.e., mixed-type irritable bowel syndrome), expression of PPARγ in colonic mucosa decreases [50]. The studies suggest important associations between dysbiosis of commensal microbes and dysregulation of PPARγ in irritable bowel syndrome [51].

Mice artificially infected with *Trichinella spiralis* represent an in vivo irritable bowel syndrome model. In this disease model, beneficial *Akkermansia* decreased and pathogenic bacteria, such as *Escherichia/Shigella*, increased [51]. In the colonic tissue of these mice, expression of tumor necrosis factor (TNF)-α markedly increased, whereas the levels of PPARγ and a tight junction protein (occludin) decreased. Pretreating these mice with a prebiotic blend (containing fructo-oligosaccharide, galacto-oligosaccharide, inulin, and anthocyanins) ameliorated *Trichinella spiralis*-induced changes in dysbiosis and dysregulation of TNF-α, PPARγ, and occludin [51]. Although the underlying mechanism is not well defined, proteomics analysis of this study revealed that the beneficial effects of the prebiotic blend are associated with a PPARγ-mediated pathway [51]. 

Water-avoidance stress in mice is another in vivo model of irritable bowel syndrome. This type of stress reduces the expression of intestinal nucleotide-binding oligomerization domain protein-like receptors, pyrin-domain containing (NLRP)-6 [52]. NLRP6 is an inflammasome that is important for maintaining gut microbial homeostasis. Therefore, water-avoidance stress-induced reduction of NLRP6 causes an increased ratio of the Firmicutes to Bacteroidetes [52], a pattern similar to that seen in irritable bowel syndrome patients [46,47]. Pretreating these mice with probiotic supplementation containing *Bifidobacterium bifidum*, *Lactobacillus acidophilus*, and *Streptococcus faecalis*, or treating with rosiglitazone (a PPARγ agonist) increased NLRP6 and reversed stress-induced intestinal inflammation [52,53,54].

**Figure 2:**
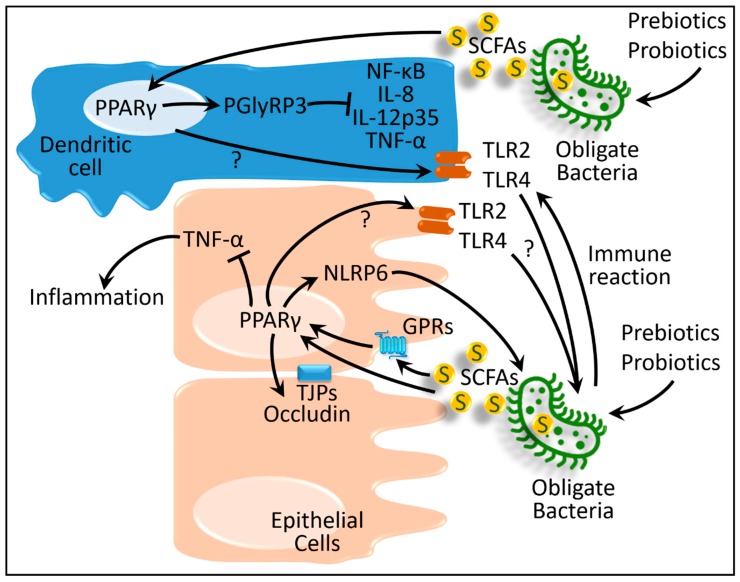
Schematic presentation of interactions between host PPARs and gut microbiota in gastrointestinal disease. GPRs, G-protein-coupled receptors; IL, interleukin; NF-κB, nuclear factor κB; NLRP6, nucleotide-binding oligomerization domain protein-like receptors, pyrin-domain containing 6; PGlyRP3, peptidoglycan recognition protein 3; PPARγ, peroxisome proliferator-activated receptor γ; S and SCFAs: short-chain fatty acids; TJPs, tight junction proteins; TLR, Toll-like receptor; TNF-α, tumor necrosis factor α. ‘?’ denotes the contradictory or ambiguous evidences in the literatures. Black lines ending in arrowheads denote activation and lines ending in bars represent inhibition.

Similar to irritable bowel syndrome, the etiology of inflammatory bowel diseases is unknown. However, it is assumed that, in genetically susceptible individuals, dietary ingredients either directly [55] or through microbiota [56,57] interact with intestinal immune cells and epithelial cells. These interactions pertain to the inflammatory responses. Supplementation of fructo-oligosaccharide to patients with Crohn’s disease, a variant of irritable bowel syndrome, stimulates the growth of beneficial *Bifidobacteria*. Fructo-oligosaccharides inhibit intracellular transcription factors, such as nuclear factor κB and, thus, promote IL-10 and inhibit IL-12 expression in intestinal dendritic cells [57]. Peptidoglycan recognition protein 3 (PGlyRP3), a member of the PGlyRP family, acts as a pattern recognition molecule in the intestinal epithelium [58]. PGlyRP3 reduces expression of proinflammatory IL-8, IL-12p35, and TNF-α cytokines. PPARγ is a transcriptional activator of PGlyRP3 [59]. As mentioned above, *Bifidobacteria* produces short-chain fatty acids, such as butyrate, which are ligands for PPARs. Therefore, it is conceivable that oligosaccharide-stimulated *Bifidobacteria* growth enhances expression of PPARγ, and its targets, such as PGlyRP3, reduce proinflammatory cytokines. Additionally, PPARγ supports maintenance of several commensal bacteria such as *Candida albicans* and *Bacteroides fragilis*. In this process, PPARγ activates β-defensin-1-mediated immunity in Crohn’s disease [60], which constitutes another intestinal anti-inflammatory mechanism. Presumably, all the above mentioned PPAR-mediated mechanisms are protective against irritable bowel syndrome (Figure 2) [54,55,61].

In intestinal epithelial cells and dendritic cells, the expressions of pattern recognition receptors, such as Toll-like receptors (TLR)-2 and -4, along with some inflammatory cytokines (IL-18 and IL-1β), are increased in both irritable bowel syndrome and inflammatory bowel diseases [50,55,57,62]. Oligosaccharide treatment increases TLR2 and TLR4 in inflammatory bowel disease [55,57]. It was, therefore, postulated that TLRs play some protective roles by facilitating recognition of local microbiota and enhancing homeostasis along with providing cytoprotective effects [50,63]. The combined anti-inflammatory effects produced by PPARs, along with the TLR-mediated cytoprotective effects, might be beneficial for inflammatory bowel diseases; however, further clarification is required to explore the microbiota–PPAR–TLR interactions in inflammatory bowel diseases and irritable bowel syndrome.

All the abovementioned studies in this section postulate that PPARs (1) activate the intestinal epithelial tight junction protein occludin, (2) increase intestinal NLRP6 to reverse intestinal inflammation, (3) increase anti-inflammatory PGlyRP3 and decrease proinflammatory cytokines such as, IL-8, IL-12p35, and TNF-α, and (4) possibly activate TLR2 and TLR4 to a certain level to allow the growth of the facultative microbiota. These balanced pro- and anti-inflammatory actions of PPARs thereby control bowel disorders (Figure 2).

### 4.2. Effects of Gut Microbiota and PPAR Interactions in Obesity and Metabolic Syndrome

Metabolic syndrome (MetS) is a complex of diseases broadly comprising dyslipidemia, insulin resistance, and type 2 diabetes mellitus. Obesity is a major predisposing factor of MetS [17,20]. Adipose tissue is the specialized organ that efficiently deposits extra energy as fat during nutritional excess and is used for releasing energy in times of nutritional deprivation. However, adipose tissue also acts as an active endocrine organ, maintaining whole-body energy homeostasis by releasing many adipocytokines such as adiponectin. However, in times of continuous nutritional overload, adipose tissue becomes hypertrophic and hyperplastic [64,65]. The hypertrophic adipocytes release inflammatory adipocytokines, such as IL-6, TNF-α, etc., creating a low-grade inflammatory milieu [20,66]. This inflammatory state is considered a major predisposing factor for obesity-induced pathogeneses [17,20].

In addition to adipose tissue dysfunction, dysbiosis of intestinal microbiota can initiate a similar inflammatory milieu that predisposes a person to metabolic disorders [67]. As mentioned in different instances within this review, a notable feature of obesity-induced intestinal dysbiosis is an increase in Firmicutes phyla and a decrease in Bacteroidetes phyla [68,69]. However, Firmicutes are Gram-positive and Bacteroidetes are Gram-negative bacteria. Therefore, a low abundance of Gram-negative Bacteroidetes in obesity should reduce serum lipopolysaccharide levels, an endotoxin mostly produced by Gram-negative bacteria. Thus, the causative role of microbiota in low-grade inflammation, as seen in obesity, cannot merely be explained by the reduction in Bacteroidetes [67]. In type 2 diabetes patients, in addition to Firmicutes, lipopolysaccharide-producing *E. coli* (phylum Proteobacteria) also increases [69]. This latter species may contribute to enhanced systemic inflammation of the intestine. In this dysbiosis, gut permeability is also impaired due to reduced expression of enterocyte tight junction regulators such as zonula occludens-1 and occludin. As a result, lipopolysaccharide can be easily transported in the circulation system [70].

In this regard, some chemical compounds aimed at activating PPARγ were shown to reverse high-fat diet-induced dysbiosis of the gut microbiota. *Salvia miltiorrhiza*, a plant-derived tanshinol borneol ester (Danshensu Bingpian Zhi, DBZ), is a synthetic derivative of natural compounds. DBZ is an activator of PPARγ; however, the potency is lower than thiazolidinedione. In a high-fat diet-induced, obesity-related MetS mouse model, DBZ reduced the high-fat diet-induced increased Firmicutes-to-Bacteroidetes ratio. In MetS, the abundance of Firmicutes population upregulates the intestinal absorption and metabolism of fatty acids, which increases the intestinal lipid droplet number [71]. Alternatively, in lean animals, a low abundance of Firmicutes or some other species decreases dietary carbohydrate processing, inhibits excess energy absorption, and reduces lipogenesis [72]. DBZ increases *Akkermansia*, which are beneficial bacteria belonging to phylum Verrucomicrobia, and suppresses high-fat diet-induced harmful bacteria, including *Helicobacter marmotae*, Odoribacter, and Anaerotruncus. Therefore, DBZ-mediated PPARγ activity improves high-fat diet-induced dysbiosis, weight gain, and insulin resistance in diabetic mice [73].

**Figure 3:**
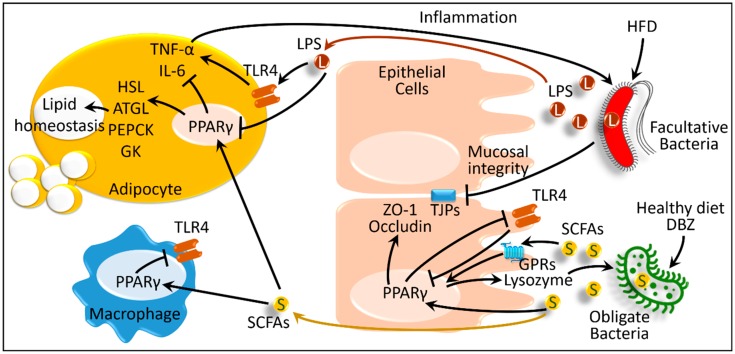
Schematic presentation of interactions between host PPARs and gut microbiota in obesity and metabolic syndrome. ATGL, adipose triglyceride lipase; DBZ, tanshinol borneol ester Danshensu Bingpian Zhi; GK, glycerol kinase; GPRs, G-protein-coupled receptors; HFD, high-fat diet; HSL, hormone-sensitive lipase; IL, interleukin; L and LPS, lipopolysaccharide; PEPCK, phosphoenolpyruvate carboxykinase; PPARγ, peroxisome proliferator-activated receptor γ; S and SCFAs: short-chain fatty acids; TJPs, tight junction proteins; TLR, Toll-like receptor; TNF-α, tumor necrosis factor α; ZO-1, zonula occludens-1. Black lines ending in arrowheads denote activation and lines ending in bars represent inhibition. Yellow and red arrows depict absorption of short-chain fatty acids and lipopolysaccharide, respectively.

As a mechanism, gut microbes convert dietary fibers such as pyruvate, succinate, and lactate into short-chain fatty acids, such as acetate, butyrate, and propionate. Fatty acids bind with specific G-protein-coupled receptors, such as GPR109A, GPR41, and GPR43, or can directly activate nuclear receptors such as PPARs and initiate several biological actions [32,74]. In the intestine, butyrate suppress proinflammatory macrophages, propionate enhances satiety, and acetate affects adipose tissue, brain, and liver, and improves metabolic effects [32]. Short-chain fatty acids, even acetylate and methylate histone, modify the chromatin structure and epigenetically regulate genes involved in PPAR signaling and diabetes mellitus [5]. Another line of evidence suggests that obese subjects with higher insulin resistance and fasting serum triglyceride levels show lower bacterial gene abundance than lean subjects (379,436 and 567,499 genes for obese and lean subjects, respectively) [75]. Presumably, the low abundance of microbial genes results from a reduction in butyrate-producing bacteria [76]. Thereby, high-calorie diets reduce microbial short-chain fatty acid production, whereas energy-restricted diets improve the gene richness in obese subjects, along with reducing overall obesity trends of fat mass, circulating cholesterol and, to some extent, inflammation [5,76]. Through these mechanisms, short-chain fatty acids produced by the microbiota alter the metabolic state through PPAR signaling (Figure 3).

However, not only the variations in species, but also different strains of bacteria play diverse roles in obesity. *Helicobacter pylori* is a dominant gastric microbiota. Excessive presence of *H. pylori* in the stomach and duodenum commonly predisposes a person to peptic ulcer disease [77]. However, a strain containing mutated cytotoxin-associated gene pathogenicity island (cag PAI) is associated with increased PPARγ activation and upregulation of its target genes (i.e., CD36 and fatty-acid-binding protein 4) in a mouse model. Mice infected with this strain show enhanced influx of regulatory T cells into white adipose tissue during obesity. However, humans are always colonized by both strains [78]. The cag PAI mutated strain provides protection against metabolic disorders by augmenting anti-inflammatory responses [79]. Therefore, disappearance of gastric *H. pylori* due to consumption of antibiotics may, at least in part, contribute to enhanced epidemics of obesity and other related metabolic disorders [79].

The assumption about dysbiosis in the context of MetS is partly TLR4-centered. In MetS, inflammasomes (i.e., lipopolysaccharides) activate TLR4 in adipocytes and inflammatory cells, thus inducing the inflammatory milieu in obesity [70]. In addition to adipose tissue, in MetS, intestinal epithelial TLR4 dysregulation plays a vital role. In an experimental mouse model, inactivation of TLR4 only in the intestine, but not the whole body, instigates MetS. Intestinal TLR4 deficiency downregulates PPARγ and the antimicrobial peptide lysozyme expressions in the intestine [80]. Due to the lack of antimicrobial lysozyme, the bacterial population also alters; thus, microbial genes involved in the metabolism of lipids, amino acids, and nucleotides are also dysregulated. PPARγ deficiency itself predisposes a person to MetS. However, the PPARγ agonist (i.e., rosiglitazone) ameliorates the dysbiosis and the metabolic abnormality [80]. Therefore, PPARγ activity reverses the pro-inflammatory effects of intestinal epithelial receptors, such as TLR4, thus maintaining intestinal microbial homeostasis as an additional mechanism to ameliorate MetS.

Taken together, high-caloric diets alter the intestine microbial homeostasis plausibly through the TLR4–PPARγ-mediated pathway. This alteration leads to (1) an increase in the inflammasome (lipopolysaccharide)-producing microbial population, and (2) a decrease in the short-chain fatty-acid-producing microbes. As a result, systemic inflammation increases while short-chain fatty-acid production decreases. Short-chain fatty acids activate PPARs in various organs, including adipose tissue, to modulate lipolytic genes such as hormone-sensitive lipase, adipose triglyceride lipase, and lipogenic genes such as phosphoenolpyruvate carboxykinase and glycerol kinase; they also help properly metabolize and utilize fat [32,65,81]. However, due to a lack of short-chain fatty acids, PPAR activity also decreases, which causes accumulation of excess fat in obesity. Therefore, restoration of the microbial population restores the whole pathway and eventually activates PPARs, thus improving obesity (Figure 3).

### 4.3. Effects of Gut Microbiota and PPAR Interactions in Liver Diseases

In western and Middle Eastern countries, non-alcoholic fatty liver disease is the most common type of chronic liver disease [82]. The major predisposing factors of non-alcoholic fatty liver disease are an excessive supply of fatty acids in the liver and impaired disposal of lipids from the liver. Obesity with dyslipidemia is typically associated with non-alcoholic fatty liver disease [82,83,84]. In obesity, excessive fatty acids are released into circulation via lipolysis of triglycerides from the adipocytes. Absorption of dietary fatty acids from the intestine increases, as does de novo lipogenesis in the liver. Thus, the supply of lipids to the liver increases [20,85]. The excessive surge of lipids eventually provokes endoplasmic reticulum stress in the hepatocytes, thus leading to hepatocellular injury [86]. In parallel, mitochondrial β-oxidation and esterification, which forms triglycerides, are also impaired in non-alcoholic fatty liver disease [86].

As primary metabolic energy substrates, carbohydrates and fatty acids are excessively present in obesogenic diets. Fat- and carbohydrate-restricted diets emerged as effective dietary interventions for non-alcoholic fatty liver disease [86]. In the process, the intestinal microbiota plays some critical roles. In obese subjects with non-alcoholic fatty liver disease, when maintained on isocaloric low-carbohydrate diet for seven days, the numbers of folate-producing *Streptococcus* and *Lactococcus* markedly increase. As a result, serum folate level also increases [87]. Folate upregulates the expression of genes involved in folate-dependent one-carbon metabolism (i.e., β-hydroxybutyrate) and through some intermediate products, folate generates glutathione. As an antioxidant, glutathione maintains β-oxidation. Additionally, hepatic expression of PPARα and its downstream genes involved in β-oxidation increases in these obese subjects [87]. Although humans require daily folate intake, it can be produced from consumed milk products via the activity of *Streptococcus*, *Lactococcus*, and *Bifidobacterium* species [88]. Thus, low-carbohydrate diets improve non-alcoholic fatty liver disease, at least in part, by increasing folate-producing bacterial population in the gut and folate-induced PPARα-mediated β-oxidation in the liver.

**Figure 4:**
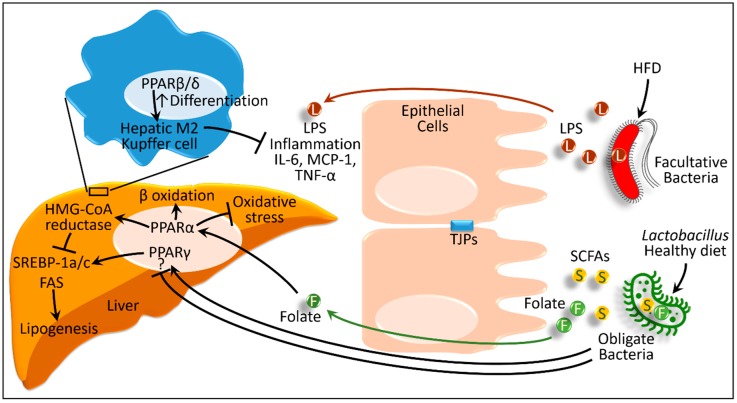
Schematic presentation of interactions between host PPARs and gut microbiota in obesity and metabolic syndrome. F, Folate; FAS, fatty acid synthase; HFD, high-fat diet; HMG-CoA, β-hydroxy β-methylglutaryl co-enzyme A; IL, interleukin; L and LPS, lipopolysaccharide; MCP-1, monocyte chemoattractant protein 1; PPAR, peroxisome proliferator-activated receptors (α, β/δ, and γ); S and SCFAs: short-chain fatty acids; SREBP, sterol regulatory element-binding protein; TJPs, tight junction proteins; TNF-α, tumor necrosis factor α. ‘?’ denotes the contradictory or ambiguous evidences in the literatures. Black lines ending in arrowheads denote activation and lines ending in bars represent inhibition. Green and red arrows depict absorption of folate and lipopolysaccharide, respectively.

High-fat diet-fed mice models usually show liver steatosis associated with an increase of phyla Firmicutes, Proteobacteria, and Verrucomicrobia [89,90]. Members of Proteobacteria express endotoxins and produce lipopolysaccharides, whereas members of Firmicutes (*Erysipelotrichaceae*) and Verrucomicrobia (*Akkermansia muciniphila*) degrade the mucus barrier [91]. Together, this form of dysbiosis results in increased circulating inflammatory mediators, such as TLR4, IL-6, and TNF-α, along with endotoxemia [92,93,94]. Bacteroidetes, *Lactobacillus*, and *Parabacteroides* populations decrease in such pathogeneses [90]. *Lactobacillus* (*Lactobacillus rhamnosus* GG and *Lactobacillus platarum* WCFS1) show protective effects against non-alcoholic fatty liver disease [90]. In this study, *Lactobacillus* strains reversed the high-fat diet-induced dysbiosis such that not only *Lactobacillus*, but also Bacteroidetes and *Parabacteroides* populations increased. This alteration of symbiosis was associated with reversal of the epithelial barrier integrity and improved intestinal barrier function through upregulated expression of intestinal tight junction protein occludin and zonula occludens-1. The levels of endotoxemia and hepatic steatosis were also reversed by the *Lactobacillus*. In the presence of the anthraquinone from a herb named *Cassia obtusifolia* L., all these improvements were further augmented [90]. The authors showed that these treatments enhanced hepatic expressions of PPARα while decreasing the expressions of PPARγ, β-hydroxy β-methylglutaryl co-enzyme A (HMG-CoA) reductase, and sterol regulatory element-binding protein-1c (Figure 4) [90].

Similarly, continuous consumption of a fructose-rich diet induces non-alcoholic fatty liver disease-like changes and insulin resistance in animal models. *Lactobacillus* (*Lactobacillus casei* and *Lactobacillus reuteri* GMNL-263) treatment activates hepatic PPARγ and attenuates inflammatory mediators, such as TLR4, IL-6, and TNF-α, which subsequently attenuates steatosis [93,94]. *Lactobasillus* decreases expression of several lipogenic genes (sterol regulatory element-binding protein-1a and fatty-acid synthase). This PPARγ-mediated change caused by *Lactobacillus* has promising therapeutic potential in obesity-mediated type 2 diabetes mellitus and associated non-alcoholic fatty liver disease [94]. In this regard, Alves et al. showed that, in the hypercholesteremic mice model, symbiotic strains (*Lactobacillus paracasei* Lpc-37, *Lactobacillus rhamnosus* HN001, *Lactobacillus acidophilus* NCFM, and *Bifidobacterium lactis* HN019) and prebiotic fructo-oligosaccharides enhanced β-oxidation through PPARα, as well as reduced lipogenesis through sterol regulatory element-binding protein-1c. As a result, hypercholesterolemia-induced non-alcoholic fatty liver disease-like changes in the liver were improved [95].

*Lactobacillus* (*Lactobacillus paracasei*) also induces proliferation of hepatic F4/80^+^CD206^+^ cells (macrophage 2 (M2) Kupffer cells) [96]. The classical variants of macrophages (M1) activate non-alcoholic fatty liver disease, whereas the regulatory circuit that stimulates differentiation of the alternative variant (M2) suppresses steatosis. In general, the alternative activation of macrophages (M2 subgroup) accounts for anti-inflammatory effects [97]. For non-alcoholic fatty liver disease or non-alcoholic steatohepatitis, the alternative variant of macrophage (M2 Kupffer cells) is proposed to have protective effects. In the process, PPARβ/δ in Kupffer cells presumably induces the alternative activation of the M2 phenotype [98].

Not only non-alcoholic steatohepatitis, but also the anti-inflammatory effects of *Lactobacillus* (*Lactobacillus casei* Zhang) were observed in a lipopolysaccharide- and d-galactosamine-induced acute liver failure rat model [99]. In this model, *Lactobacillus* increased the expression of PPARγ and attenuated TLR2, and TLR9 triggered phosphorylation of extracellular signal-regulated kinase (ERK), c-Jun N-terminal kinase (JNK), p38, and mitogen-activated protein kinase (MAPK) and prevented intestinal injury and increased fecal *Lactobacillus* and *Bifidobacterium* levels [99].

In contrast to the abovementioned studies, germ-free mice inoculated with feces from non-alcoholic steatohepatitis patients and fed a high-fat diet showed an increase in abundance of *Lactobacillus* [100]. Each probiotic has distinct effects on lipid metabolism and, thus, differentially affects the liver and other organs. More specifically, in lean animals, *Lactobacillus fermentum* and *Lactobacillus ingluviei* cause weight gain, whereas, in obese animals, *L. gasseri* and *L. plantarum* have anti-obesity effects [101,102]. The effects can be explained by the fact that each microbiota has its own capacity to metabolize nutrients and release substrates that are absorbed and used by the body. Chiu et al. showed that the hepatic expression of PPARγ along with inflammatory markers (IL-6, monocyte chemoattractant protein 1, TLR2, TLR4, and TNF-α) increased in the mice that received microbiota from non-alcoholic steatohepatitis patients and were fed a high-fat diet [100]. This study further proves that it is important to carefully choose microbial strains when planning clinically applicable probiotic regimens.

We observe that, although the effects of PPARγ show some ambiguities, PPARγ, along with PPARα and PPARβ/δ, plays some interesting roles in the liver. Effects of endotoxin-producing microbes disrupt intestinal integrity and activate systemic inflammatory responses, which result in steatosis or steatohepatitis. As a systemic effect, some microbes, such as *Lactobacillus*, (1) attenuate the inflammatory responses, and (2) facilitate functions of PPARs in hepatic and Kupffer cells to restore hepatic pathogeneses. The microbes also enhance production of some metabolites (such as folate) and activate hepatic PPARα. PPARα activation (1) enhances β-oxidation of fatty acids, (2) decreases lipogenesis, and (3) reduces oxidative stress. Thereby, PPARs protect against non-alcoholic hepatic steatosis and steatohepatitis (Figure 4).

### 4.4. Effects of Gut Microbiota and PPAR Interactions in the Cardiovascular System

**Figure 5:**
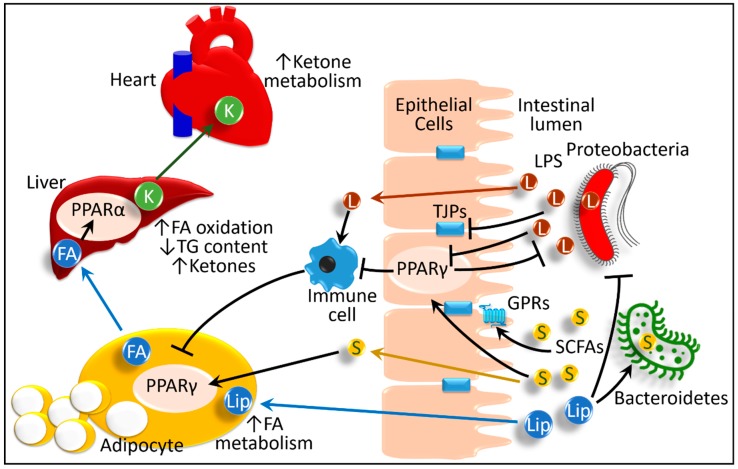
Schematic presentation of interactions between host PPARs and gut microbiota in health and disease. A healthy diet maintains a normal microbial population, that is, more beneficial short-chain fatty acid (S)-producing Bacteroidetes phyla. The short-chain fatty acids activate their respective G-protein-coupled receptors (GPRs), as well as PPARγ, in the intestinal epithelium. These processes activate tight junction proteins (TJPs) and maintain intestinal integrity. PPARγ retains the immune cell response at the physiologic level adequate to maintain microbial homeostasis. In healthy conditions, adipose tissue metabolizes lipid (Lip) and sustains serum fatty-acid (FA) levels through PPARγ activity. Hepatic PPARα sustains fatty acids through activating β-oxidation. The liver also produces ketone bodies (K), which are used by the heart as a source of high energy. However, high-fat diets cause dysbiosis and increase lipopolysaccharide-producing Proteobacteria phyla. Lipopolysaccharides and other inflammatory molecules activate local and systemic inflammatory responses and cause leakage of inflammatory mediators into the circulatory system. This process predisposes people to intestinal abnormalities such as inflammatory bowel disease and irritable bowel syndrome. The inflammatory response hampers lipid metabolism in adipose tissue and liver. These cause obesity, metabolic abnormalities, atherosclerosis, and non-alcoholic fatty liver diseases. Taken together, these processes account for additional mechanisms of microbiota-induced bowel diseases, metabolic syndrome, type 2 diabetes mellitus, and atherosclerosis. FA, fatty acids; GPRs, G-protein-coupled receptors; K, ketone bodies; Lip, lipids; L and LPS, lipopolysaccharide; PPAR, peroxisome proliferator-activated receptors (α and γ); S and SCFAs: short-chain fatty acids; TJPs, tight junction proteins. Black lines ending in arrowheads denote activation and lines ending in bars represent inhibition. Blue arrows represent transportation of lipids and fatty acids. Green arrow depict mobilization and yellow arrow depicts absorption of short-chain fatty acids.

The work by Mencarelli et al. [103] expanded our understanding of the effect of gut microbiota from non-alcoholic steatohepatitis on atherosclerosis using the apolipoprotein E (ApoE)-deficient mice model. This mouse model is widely used to study hyperlipidemia-associated atherosclerosis. Exposing these mice to dextran sodium sulfate caused breaking of the intestinal barrier and transition from steatosis to non-alcoholic steatohepatitis [103]. These ApoE-deficient mice showed severe atherosclerotic lesions, insulin resistance, and features of steatohepatitis. Treating these mice with VSL#3, a mixture of eight probiotic strains (*Lactobacillus plantarum*, *Lactobacillus delbrueckii* subsp. *bulgaricus*, *Lactobacillus casei*, *Lactobacillus acidophilus*, *Bifidobacterium breve*, *Bifidobacterium longum*, *Bifidobacterium infantis*, and *Streptococcus salivarius* subsp. *thermophilus*) [53] significantly attenuated the atherosclerotic plaque produced by dextran sodium sulfate. CD5^+^ B lymphocytes isolated from the spleen showed that VSL#3 reversed the dextran-sodium-sulfate-induced phenotype changes to a less inflammatory phenotype. Messenger RNA (mRNA) expression of PPARγ increased in the gut epithelium, and conditioned media collected from the growth of VSL#3 enhanced the luciferase reporter activity of PPARγ in HepG2 hepatocytes. This study suggested that probiotics regulate intestinal inflammation and protect against steatohepatitis and atherosclerosis through increasing hepatic and intestinal PPARγ (Figure 5) [103].

Similar to energy excess, in states of energy deprivation, the microbiota plays an interesting role in the heart. Fasting for 24 hours causes a shift in microbiota toward an increase in Bacteroidetes (42.3% in fasted vs. 20.6% in fed mice) and a decrease in Firmicutes (52.6% in fasted vs. 77.1% in fed mice) [104]. This pattern is the opposite from that generally observed in high-fat feeding: a decrease in Bacteroidetes and an increase in Firmicutes phyla [68,69]. Mice transplanted with microbiota collected from distal gut show higher serum β-hydroxybutyrate, a ketone ester [105], than the mice devoid of gut microbiota. During fasting, the bacteria-transplanted mice showed high hepatic triglyceride stores and higher PPARα expression in their liver, along with increased production of hepatic β-hydroxybutyrate. However, the fasting-induced ketogenic response, such as serum β-hydroxybutyrate level and hepatic expression of 3-hydroxy-3-methylglutaryl-CoA synthase, a PPARα-responsive ketogenic enzyme, is blunted in PPARα knock-out mice. These findings suggest that the effect of gut microbiota on fasting-induced ketosis involves PPARα [104]. The authors proposed that fasted mice containing gut microbiota produce and absorb more acetate, which acts as a substrate for increasing the hepatic triglyceride level. This triglyceride enhances production of ketone bodies in the liver and their release into circulation [104]. Due to its high energy demand, the heart can produce and use energy either through oxidation of fatty acids or from ketone bodies [105]. Between them, the use of ketone bodies is more energetically efficient and yields more energy. Therefore, during fasting, the higher level of ketone bodies, produced from the liver of the mice containing gut microbiota, acts as a highly efficient and ready source of energy for the heart. In this beneficial effect of gut microbiota on the heart during fasting, hepatic PPARα-mediated production of ketone bodies plays a pivotal role (Figure 5) [104].

Endothelial nitric oxide synthase and nitric oxide (eNOS–NO) signaling is vital for maintaining cardiac function, as well as for protecting against atherosclerosis. Nitric oxide, produced from the myocardium or from any extrinsic sources, regulates the heart rate and force of contraction, and also helps remodel the heart after myocardial infarction [106,107]. PPARs, in particular PPARα, activate nitric oxide production in these tissues [108]. Therefore, in some cardiovascular dysfunctions, intestinal microbiota-derived short-chain fatty acids such as butyrate presumably contribute to improving cardiovascular pathogeneses [108]. However, gut microbiota can produce some metabolites, such as trimethylamine *N*-oxide (TMAO), which are known to develop atherosclerosis, heart failure, and peripheral artery diseases [109]. As the exact mechanisms through which PPARs and the microbiota regulate or dysregulate cardiovascular functions remain controversial, further studies are required to better elucidate these pathways.

## 5. Conclusions and Perspective

In this review, we confined our discussion to limited types of diseases: those related to digestion, immunity, and metabolism. In these fields, microbiota-based therapeutic approaches show promise as remedies for many currently incurable diseases such as type 2 diabetes mellitus, non-alcoholic fatty liver diseases, irritable bowel syndrome, and inflammatory bowel disease. The concepts of microbiota-based therapeutic approaches are gradually changing: from changing dietary habits to modulating microbiota [10], to ingesting some probiotic strains, to producing beneficial short-chain fatty acids [32]. Novel concepts, such as ingesting genetically engineered bacteria to deliver specific bioactive small molecules and regulating host G protein-coupled receptors, are emerging [8]. Despite the limited clinical trials and laboratory-based findings remaining far from introducing effective therapies, the therapeutic benefits of microbiota through the modulation of PPARs remain pivotal.
